# How general practitioners decide on maxims of action in response to demands from conflicting sets of norms: a grounded theory study

**DOI:** 10.1186/s12910-019-0360-3

**Published:** 2019-05-14

**Authors:** Linus Johnsson, Lena Nordgren

**Affiliations:** 1 Department of Public Health and Caring Sciences, Uppsala University, BMC, Box 564, 751 22 Uppsala, Sweden; 2 Centre for Clinical Research Sörmland/Uppsala University, Kungsgatan 41, 631 88 Eskilstuna, Sweden

**Keywords:** General practitioners, Grounded theory, Quality of care, Ethics, professional, Family medicine, Ethical decision making, Sweden

## Abstract

**Background:**

The work of general practitioners (GPs) is infused by norms from several movements, of which evidence based medicine, patient-centredness, and virtue ethics are some of the most influential. Their precepts are not clearly reconcilable, and structural factors may limit their application. In this paper, we develop a conceptual framework that explains how GPs respond, across different fields of interaction in their daily work, to the pressure exerted by divergent norms.

**Methods:**

Data was generated from unstructured interviews with and observations of sixteen Swedish GPs (who have by definition more than five years of experience after license to practice) and family medicine residents (with less than five years of experience) between 2015 and 2017. Straussian Grounded Theory was used for analysis.

**Results:**

We found that GPs’ maxims of action can be characterised in terms of dichotomous responses to demands from four distinct sets of norms, or “voices”: the situation, the self, the system, and the profession. From the interactions between these voices emerge sixteen clusters of maxims of action. Based on the common features of the maxims in each cluster, we have developed a conceptual framework that appears to be rich enough to capture the meaning of the ethical decisions that GPs make in their daily work, yet has a high enough level of abstraction to be helpful when discussing the factors that influence those decisions.

**Conclusions:**

Our four-dimensional model of GPs’ responses to norms is a first step toward a middle-range theory of quality from GPs’ perspective. It brings out the complexity of their practice, reveals tensions that easily remain invisible in more concrete accounts of their actions, and aids the transferability of substantive theories on GPs’ ethical decision making. By explaining the nature of the ethical conflicts that they experience, we provide some clues as to why efforts to improve quality by imposing additional norms on GPs may meet with varying degrees of success.

## Background

The work of general practitioners (GPs) is infused by a wide array of norms from various sources. Two particularly influential movements are evidence based medicine [1] and patient-centredness [2]. More recently, interest has reawakened within the profession for virtue ethics [3]. As the precepts of these movements differ widely, it is not clear that their demands are reconcilable. The actual aims of GPs have been found to be complex, encompassing clinical, patient related, and resource related perspectives [4]. Aiding our understanding of how GPs respond to pressure from divergent norms, especially when they clash with the realities of GPs’ working conditions, is therefore an essential goal of a theory of quality from the GP’s perspective.

*Evidence based medicine* (EBM) aims to help clinicians “move beyond intuition, expertise, and expert opinion … in determining the best clinical evidence for the patient in front of them” [5]. Although the practice of EBM, according to its original formulation, presumes that use of guidelines must be integrated with knowledge of “the patient’s clinical state, predicament, and preferences,” [6] its frequent association with performance incentives has raised concerns that aspects of quality that are less well understood among policy makers might be hampered. For instance, Bower et al. found that primary care professionals were often torn between quality targets and the patient’s agenda [7]. According to Müller-Riemenschneider et al., GPs were reluctant to divert precious consultation time away from the patient’s concerns in order to perform standardised risk assessments on healthy adults [8]. In a study by Freeman and Sweeney, GPs were aware of their power to sway patients, sometimes to the point of pre-empting their decisions [1]. This insight created a tension between their perceived duty to persuade the patient to accept evidence-based treatments and respect for their autonomy.

Although *patient-centredness* is arguably one of the core values of general practice [9], seeking it is not always straightforward. According to Bensing et al., consultations in 2002 were more task-oriented and businesslike than in 1986, partly due to expectations to practice EBM, but perhaps also because of the distraction provided by computers [10]. Limited time, strong focus on EBM, and structures that impede continuity of care make it difficult to work fully within a patient-centred model [11], and GPs committed to delivering holistic care may struggle due to organisational and time constraints [12].

Social and organisational context may influence what values are actually embraced by GPs. According to Mead and Bower, contextual factors such as teamwork and role substitution may reduce doctors’ opportunities for getting to know their patients, and thus their “ability to attend to the more ‘idiographic’ aspects of patient-centred care,” that is, those that pertain to the understanding of individuals as opposed to groups of people [2]. Braunack-Mayer found that professional virtues were mentioned almost exclusively in the accounts of rural GPs, who work in small group practices or even alone as the town’s only doctor. The author concludes that the ability to articulate professional virtues depends on one’s commitment to and experience of community-based general practice in a context where continuity, accessibility and comprehensiveness have arisen out of necessity [13].

It should be clear at this point that a shared “ethics of general practice” cannot be taken for granted, and that attempts to improve quality by applying norms deductively may meet resistance from GPs unless one properly understands and addresses their concerns. There is therefore a need for middle-range theories that explain the ethics of GPs not just in relation to particular disease- or process-related activities, but across all domains of interaction. Understanding the complexity of their practice requires a coherent account of the values at stake, the nature of the conflict between those values, and the diversity of responses.

This paper is the first in a planned series in which we explore quality from the GP’s perspective. We will outline a sub-process in which pertinent values are resolved through selection of a *maxim,* that is, a practical principle of action [14]. Our focus is here on the *maxims* category, around which we build our conceptual framework. We believe that this framework will prove a useful building block in future studies of the relationships between the ethical decisions that GPs make, the actions that they take to further their chosen ends, and the final outcomes of the resulting interactions.

## Methods

Given our aim to develop a theory and our belief that hypotheses can be meaningfully tested through qualitative inquiry, we decided on a Straussian Grounded Theory (GT) approach [15].

### Sources of data

Our data comprise observations and interviews with Swedish primary care physicians. To be eligible, informants had to have a commitment to family medicine. Our target population therefore included, besides actual GPs (in Swedish, “specialist i allmänmedicin”), who have by definition concluded a five-year specialist training programme after license to practice, also doctors currently undergoing such a programme, commonly referred to as family medicine residents (“ST-läkare i allmänmedicin”).

The informants were recruited in 2015–2017. Recruiting commenced in the vicinity of our base of operations. We approached potential informants face-to-face during professional meetings and workshops, taking care to include both men and women with varying lengths of experience. Those that expressed interest were formally invited by email.

After the first six informants, we switched to strategic sampling. We hypothesised that GPs’ experiences might differ depending on demographics, number of staff, models of ownership, and research experience. This had implications for our recruiting strategy. We mingled at conferences and spread the word through our networks, looking specifically for diverging contexts. We recruited five informants in this manner. The last five informants were recruited through snowballing.

### Characteristics of sites and informants

The eleven included sites (healthcare centres) were located in urban areas, middle-density communities, and sparsely populated areas (less than ten citizens per km^2^) in four counties, from Southern to Northern Sweden. Two had private owners. The number of patients served per site ranged from 1,500 to well over 30,000, and the number of doctors employed from three to several dozen. Of the sixteen informants, five were family medicine residents. Of the eleven GPs, eight had worked more than fifteen years in a primary care setting.

### Data generation

We conducted one-to-three-day field trips during which one or several informants were observed and interviewed. We mostly observed doctor–patient encounters, but also meetings with peers and other staff. All involved patients consented orally. While observing, we took field notes and fleshed them out later in memos. The interviews, which lasted for 24–71 min (median 53), were unstructured but focused on the GPs’ thoughts on the observed encounters. They were audio recorded and transcribed verbatim. With one exception, each informant was observed before the corresponding interview.

### Analysis

Analysis and data generation ran in parallel throughout the project. A custom computer application was developed to aid analysis. Among the methods used, constant comparisons, coding, and memoing were most crucial.

#### Sensitivity and openness

LJ has several years of first-hand experience from general practice. We believe that this has significantly reduced the time necessary to develop our sensitivity to the informants’ concerns. As a bioethicist, he is familiar with concept analysis, but has no previous experience of qualitative research. LN has extensive experience of qualitative research, including Grounded Theory, and has supervised healthcare professionals doing research in their own working environments. She has little experience of primary care, thus contributing to openness.

To assist the reader in judging our work, we will presently describe in detail some of our procedures.

#### Construction of events

The unit of analysis was *events*. An event is here defined as a piece of data that can be thought of as a story, complete with a setting, plot, interaction, and resolution. In our material, events were entangled in three different ways. First, a complex encounter might comprise several, often intertwined, events. Second, whenever an informant reflected during the interview on what had happened during the corresponding observation, the same event would be referenced in both texts. Third, as interviews tended to weave back and forth, descriptions of a single event might be scattered across a text. To untangle the events, we first split each text into fragments, each referring to no more than one event, and thereafter recombined the fragments so that all data pertaining to an event could be assessed side-by-side. This procedure yielded a total of 471 events.

#### Triangulation

The practice of using several different kinds of sources of data is commonly encouraged in GT research because it increases the credibility of one’s interpretations. During each interview, we actively sought the informant’s thoughts on what had taken place during the observation and why they had acted as they did. By analysing observations and interviews side by side, we were able to spot instances where the informant was claiming to do one thing while in practice doing something else, and to interpret the meaning of observed events in the light of the informant’s reflections. Our chances of rejecting false hypotheses were thereby increased.

#### Constant comparisons

New data were consistently interpreted by comparing them to previously generated data. Our growing familiarity with the data gradually enabled us to recall similar events. Because similarities and differences between events form the cornerstones of our conceptual framework, our concepts are firmly anchored in what we have heard and observed rather than emerging from our pre-understanding.

#### Memos

Throughout the project, we recorded first impressions, thoughts, questions, hypotheses and ideas in memos (1,615 to date). Those written on events helped us to sensitise ourselves to the data and to develop our thoughts. Later, more theoretically guided memos helped us judge how concepts and processes reflected reality, or to set up hypotheses to be tested later. Concept-related memos gradually evolved into definitions that could be compared in order to spot inconsistencies or gaps in the framework.

#### Open coding

We tagged each event with concepts that together expressed its meaning in general terms, thus adding concepts on a low level of abstraction to the code base. We returned to this step several times during analysis, in particular each time new data had been generated.

#### Crosscutting concepts (axial coding)

Maxims (sub-concepts in the *maxims* category) were continuously examined for differences that could be represented in terms of properties and dimensions (property values). Each property came to represent a “voice,” and its dimensions the possible responses to that voice. Attributing a dimension to a maxim thus signified its response to the corresponding voice. Based on the dimensions attributed to them, maxims were grouped into “clusters” that represented response patterns on a higher level of abstraction. Each voice was also associated with a category, with each sub-concept representing a particular demand emanating from that voice.

Candidate properties were tested against three criteria of usefulness: that they be *individually relevant* (say something important), *orthogonal* (independent of each other), and *jointly exhaustive* (able to explain each encounter). Preliminary definitions of maxims were suggested early and evolved over time in the light of attributed dimensions. Five to ten events were usually required to produce a theoretically sound definition. Maxims that were becoming ubiquitous (approaching 15–20 events) or seemed out of place among their “siblings” in a cluster were questioned. In parallel, clusters were defined through a bottom-up approach.

#### Selective coding

As our code base grew and evolved, the data was re-coded multiple times. Separate selective coding runs were required for different sub-processes or when testing new concepts against the data. Many concepts and properties were discarded at this point because they did not fit the data, failed to express important enough distinctions, or contributed little to understanding. Analysis continued until clusters were homogeneous enough to coherently express core ideas and rich enough to explain the contained maxims.

## Results

In our emerging theory, the ethical aspect of the professional practice of GPs is represented in a sub-process of *deciding on a maxim of action* in which demands from different sets of norms are weighed and negotiated. Although our theory may have normative implications, it is not in itself a normative theory. Rather, whenever we mention “norms” in what follows, we refer to those that we have found in our data. In other words, our theory is one of descriptive ethics, grounded in actual morals rather than moral philosophy.

The sub-process that we describe here does not address how the entrant norms are “processed,” cognitively or otherwise; rather, the rationale for each action is contained in its maxim. Crucially, mere intentions do not count as maxims; by definition, maxims are always reflected in action.

Far from being only occasionally engaged in, ethics was a core activity of GPs. A decision whether or not to begin an investigation, for instance, would involve complex judgments on potential benefits and harms. Other times, GPs would express frustration over mandatory questionnaires displacing patients’ concerns. Some decisions had moral residues, as when the GP would run late due to an unexpected and alarming lab result requiring attention. Once we had become aware of their importance, ethical decisions could be spotted in virtually every encounter. We are convinced that they precede, logically and temporally, any strategies or techniques that GPs might employ to further their chosen ends.

In the following section, we will define four different “voices” from which the demands originate. Although a detailed account of each voice falls outside the scope of this paper, we will try to circumscribe them enough to establish them within the sub-process. We will then move on to consider pairwise interactions between voices. Because the complexity of a multidimensional model grows exponentially with the number of interacting voices, we dichotomised the responses to each voice as “positive” or “negative.” Lastly, we will present the complete four-dimensional model and provide examples of maxims in each of the sixteen clusters.

### The four voices

From our data emerge four categories that represent discrete sets of norms, each of which suggests a particular line of action, as if speaking in its own “voice.” Based on the kinds of arguments that they tend to present in support of their suggestions, we have opted to call them the *profession*, the *system*, the *situation*, and the *self*. Our terminology bears considerable resemblance to some theories of professional practice [16, 17]. We must emphasize, however, that although deduction has been crucial in validating our findings, the voices were discovered in our data rather than being defined a priori in some analytical scheme. The following subsections will detail how each voice was conceived. In general, observed conflicts between norms were paramount to this endeavour.

**Fig. 1:**
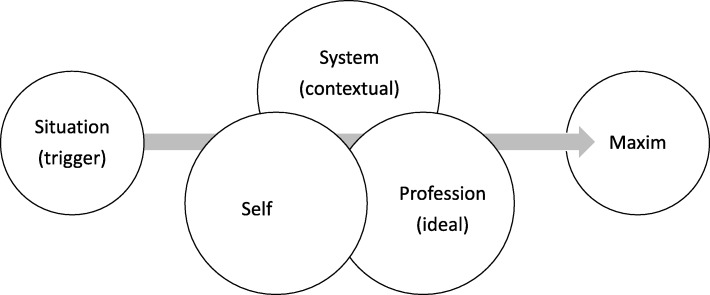
The sub-process of deciding on a maxim of action is triggered by a demand voiced by the situation itself and modified by contextually embedded norms dictated by the system, professional ideals, and the needs of the self

Figure 1 outlines how the four voices converge in the sub-process. An example of how one might begin to analyse an encounter through them is provided in Table 1.

**Table 1:** Examples of demands that may emanate from the four voices when the patient brings a lengthy list of complaints, questions and problems to their annual diabetes care review

Situation	“Answer all questions”
Self	“Avoid cognitive overload”
System	“Assess the risk factors”
Profession	“Give time to those in need”

#### The situation

The *voice of the situation* was discovered when we tested a category for capturing the overarching “plot” of the encounter. Although we came to realise that nothing short of a sub-process would suffice for that purpose, the original category was kept and remodelled into representing *triggers* – significant changes in the situation that compel the GP to take action. The change was typically, but not always, precipitated by the presence of a *thou* to which the GP must answer. Examples of imperatives stemming from the voice of the situation include: *minimise your discomfort, answer all questions, allay your fears, discuss the alternatives, facilitate your narrative, provide a quick fix, get you out of your quandary,* and *be open to alternative ideas.*

Triggers typically occur in the beginning of an encounter or, less obviously, whenever crucial pieces of information, opinions or wishes are revealed. Each trigger marks the beginning of an event. As triggers can rarely be controlled by the GP, other sub-processes may still be running, and so several sub-processes may come to run in parallel during part of or throughout the encounter.

The GP’s range of responses can be dichotomised as either *responsive* or *detached*.

##### Responsive

Striving for proximity to the situation and accepting its contingencies and idiosyncrasies, the GP is prepared to assign lesser priority to preconceived demands and agendas.

##### Detached

The particulars of the situation hold little sway over the GP, who instead seeks a birds-eye view that allows them to distribute their efforts to satisfy more predictable demands.

#### The self

Just like demands of the situation, demands of the *self* spring directly from the “micro” level of interaction. We first discovered this voice when analysing the differences between self-effacement and careful manoeuvring. It was not until several other distinctions had been made, however, that we were able to formulate its core idea: that it champions the needs that the GP has *in virtue of being human* (as opposed to their professional or functional needs). Demands of the self express the GP’s need for self-preservation, achievement, and acknowledgement. Examples include limiting one’s responsibilities, conserving one’s resources, securing some breathing space, tying up loose ends, averting criticism, putting one’s mind at ease, and gaining appreciation from patients and peers.

The GP’s responses can be dichotomised as either *self-conscious* or *other-regarding:*

##### Self-conscious

The GP takes into account what furthers their own interests or position. The motive need not be ultimately egoistic because protecting certain assets (not least the goodwill of others) might be crucial to long-term effectiveness and efficiency. Depending on what else is at stake, the outward expression might be one of unease, deference, or defensiveness.

##### Other-regarding

What furthers the GP’s own interests or position is notably absent from the equation. The GP either perceives no imminent threat or prioritises other concerns, thus becoming potentially more perceptive to other demands. Responses in this cluster range from laid-back complacency to passionate self-effacement.

#### The system

The informants frequently – and often emotionally – spoke of “Them” being in control of their working conditions. The “They” – or less abstrusely, the *system* – can be construed as a powerful entity with interests and desires of its own such as *mastering reality* (by assembling and structuring information), *optimising processes* (standardising, promoting teamwork, and substituting roles), *empowering patients* (by granting them rights to service, information and decision making) and *fixing health problems* (making the patient satisfied, commodifying health, and cultivating a public image). Although the GPs mostly perceived the system as benevolent, supportive, and in pursuit of universal goods, its demands were sometimes regarded as incoherent (for instance, “satisfy the customer while conserving resources”) or even questionable at face value (“prolong life in spite of side-effects”). Of course, even agreeable aims were occasionally eschewed when they conflicted with the demands of some of the other three voices.

We dichotomise the possible responses to the demands of the system as either *loyal* or *unfettered*, as follows:

##### Loyal

The GP acts in accordance with the system’s will, aiming for efficiency by employing standardised methods, embracing teamwork and improvement initiatives, and responding to social cues so as to minimise friction.

##### Unfettered

The GP challenges the system by improvising rather than following procedure, consuming more than their share of resources, or failing to apply their authority so as to maximise production of the kinds of goods that the system seeks.

#### The profession

The last of the four voices, that of the *profession*, is also notably “larger than self.” We originally postulated it to capture prevalent ideas of an ethical “core” in the practice of GPs. Its level of abstraction increased as we realised that contextual differences restrict the kind of ethics that can be possibly shared by all GPs. It follows that in our theory, the demands of the profession do not boil down to “accessibility,” “medical effectiveness” or the like; such demands appear to be too specific, too complex, too numerous and, taken together, too contradictory to be spoken by a single voice.

Nevertheless, a unified (albeit minimalistic) account of the voice of the profession is possible. Its hallmark is the potential conflict between the ideal and the practicable: “I know that you are supposed to … but in this case I need to … ,” often with the ideal being attributed to the impersonal *you* (in Swedish, “man”). Although a discussion of its precise structure is out of the scope of this paper, an approximation can be found in three Aristotelian virtues: *honesty*, *courage*, and *justice* [17]. This illustrates that when contextual differences between encounters have been sheared off, what can be said about GPs’ ethics can mostly be said about professionalism as such.

The GPs’ responses to the demands of the profession can be dichotomised as either *idealistic* or *pragmatic*:

##### Idealistic

The GP strives for excellence by honing their professional skills while allowing themselves to be constrained by the virtues of the profession. The practice itself occupies centre stage, whereas any goods produced and rewards gained are regarded incidental and of secondary importance.

##### Pragmatic

The GP downplays the relevance of professional virtue, applying their professional skills as tools amongst others rather than considering them particularly worthy of respect. This gives the GP more leeway to select the most promising route towards preferred outcomes.

### Summary of the four voices

To sum up the four voices and their demands, we provide in Table 2 a schema for natural-language interpretation of the process of maxim selection, using the encounter from Table 1 as example.

**Table 2:**
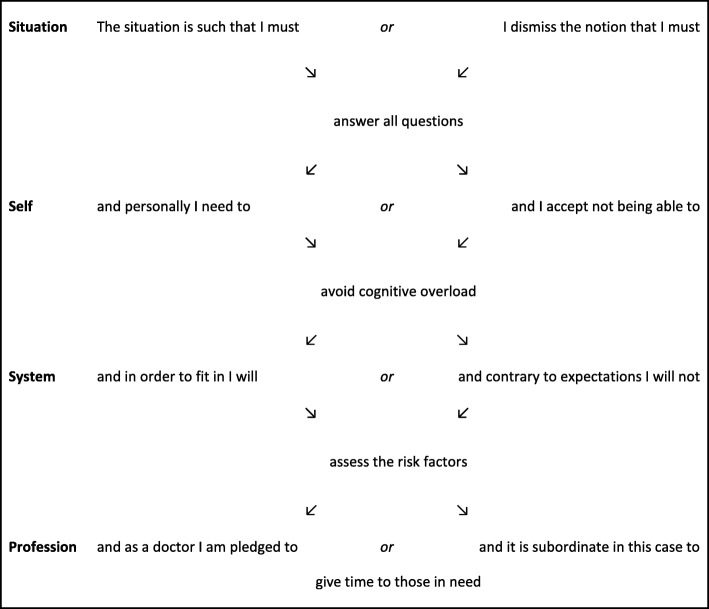
Natural-language interpretation of the sub-process of maxim selection. “Positive” responses to the demands of each voice are found to the left, and “negative” responses to the right. Responses are independent, yielding a total of 2^4^ = 16 possible paths. GPs vary, to a greater or lesser degree, their responses depending on the specifics of the situation

### Two-dimensional planes

Theoretically, four orthogonal voices interact in a total of six two-dimensional planes. Given that responses to each voice can be dichotomised, each plane assumes the form of a two-by-two matrix of clusters. Although we did explore all six planes and gained theoretical insights from each, only the most intuitive two will be presented here for brevity. The present section serves as a springboard towards understanding the fully fledged, four-dimensional space of ethical decision making.

#### The situation and the self

**Fig. 2:**
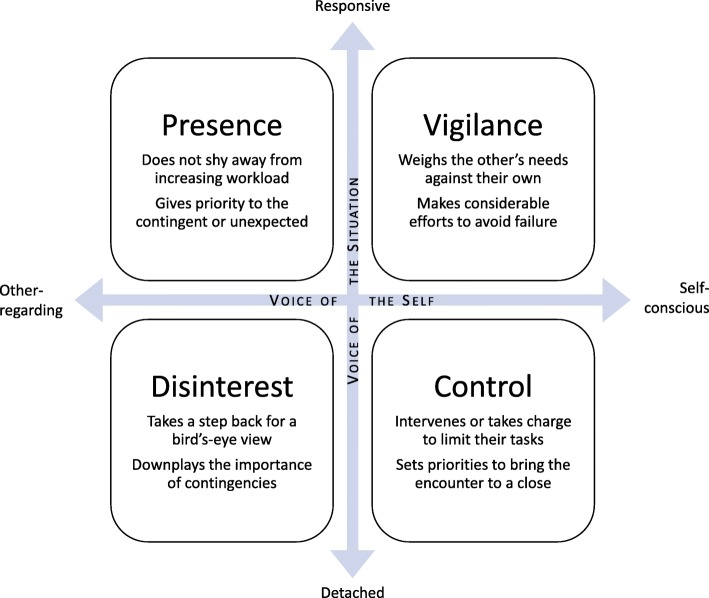
The four clusters of maxims resulting from the “micro” level interaction between the demands of the situation and the demands of the self

The four clusters of maxims that follow from the interaction between the voice of the situation and the voice of the self are outlined in Fig. 2 and further described below.

##### Vigilance

The GP strives to meet the needs of the other without sacrificing their own position or interests. Avoiding failures such as letting the other down, ending up in a hard-to-manage situation, or doing a bad job is central.

##### Presence

The GP is unselfconsciously aware of the needs of the other, labouring tirelessly even in the face of an increasing workload. The contingencies of the situation are given high priority, and so surprises and idiosyncrasies pose no particular difficulties.

##### Control

The GP feels the need to take charge in order to circumscribe their duties. In order to bring the encounter to a close, the GP may have to turn aside less important requests and look away from contingencies.

##### Disinterest

The GP observes the situation calmly from a distance, gaining a better overview at the price of intimacy. The contingencies of the situation are downplayed in favour of what can be grasped in general terms.

#### The profession and the system

**Fig. 3:**
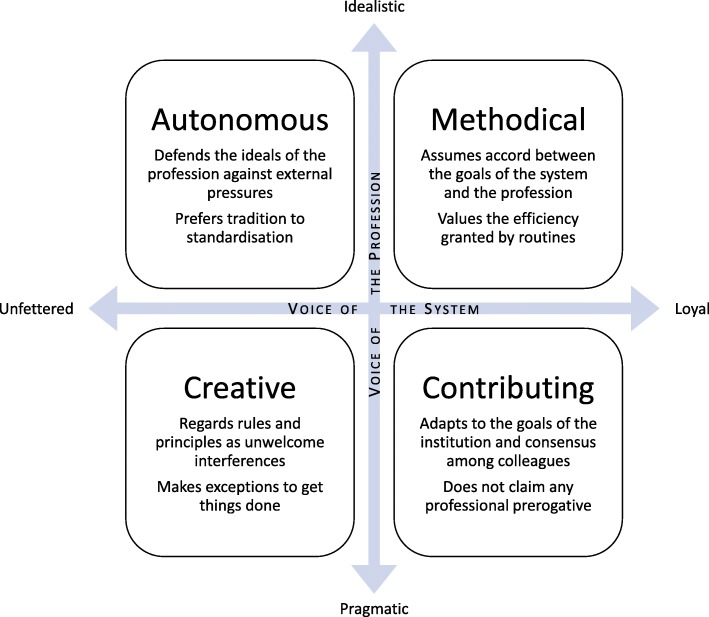
The four clusters of maxims resulting from the interaction between demands from “larger-than-self” sources (the profession and the system)

The interaction between the voice of the profession and the voice of the system also yields four clusters of maxims (see Fig. 3).

##### Methodical

The GP assumes congruence between the goals of the system and those of the profession, working strictly within those bounds and judging success by the appreciation shown by the system. Efficiency gained by following routines is valued highly.

##### Autonomous

The GP feels compelled to ignore or resist some of the system’s demands that would require sacrificing professional virtue. Because the demands of the profession are often embedded in tradition, the GP may outwardly appear to oppose change.

##### Contributing

The GP acts as an agent of the institution, adapting as necessary to align themselves with the system’s goals. By conforming to consensus rather than claiming any professional prerogative, the GP tries to ensure frictionless operation.

##### Creative

The GP defends the microcosm where truly important exchanges take place. Ideals, rules, principles and other demands emanating from the profession or context are regarded mainly as interferences.

### The four-dimensional space

Lastly, we describe the sixteen four-dimensional clusters that, on a high level of abstraction, exhaust the possible maxims that GPs may choose to act on. We provide for each a definition followed by examples of contained maxims.

#### Four clusters of vigilance

From the point of view of the voice of the system, maxims of vigilance can be further dichotomised as *keeping a low profile* (loyal) or *protecting the person* (unfettered). Depending on how they respond to the voice of the profession, vigilant maxims can be seen as either *tentative* (idealistic) or *yielding* (pragmatic).

##### Accomplish (methodical vigilance: keep a low profile tentatively)

The GP seeks to avoid failure by meeting the demands of all parties. In this delicate balancing act, the GP seeks to

- remodel requisitions that cannot be met without sacrificing professional integrity into questions that can be answered authoritatively;
- avoid misunderstandings by expressing themselves clearly and methodically; and
- downplay the importance of their skills, using – whenever allowed by prevailing consensus – examiner-independent diagnostic tools defensively.

##### Manoeuvre (autonomous vigilance: protect the person tentatively)

The GP chooses, with the other’s best interests in mind, non-standard but professionally sound courses of action, for instance by

- collaborating through informal channels whenever possible so as to avoid unnecessary paperwork;
- seeking to understand the patient’s predicament, making some concessions to show goodwill without giving up their right to veto harmful or unwarranted interventions;
- scheduling non-standard follow-up appointments to conclude unfinished businesses and ensure medical effectiveness; and
- keeping shadow records when transparency would risk harming the patient.

##### Satisfy (contributing vigilance: keep a low profile yieldingly)

The GP aims to deliver sought-after outcomes and refrains from claiming any professional prerogative. For instance, the GP

- assumes a positive attitude toward organisational changes that convey an image of a dynamic institution;
- meets the patient’s demands as long as they do not strain the system’s resources; and
- resorts to technology – barring very expensive investigations – rather than a wait-and-see approach in situations of minimal risk.

##### Suffice (creative vigilance: protect the person yieldingly)

The GP acts unconventionally to reach a good-enough outcome where their own needs are in balance with the demands of the situation. Ideals and standardised approaches are given little weight, for instance when

- giving the patient exactly what they requested (a prescription, sick note, referral, etcetera); or
- listening, in cases of medical uncertainty, for zebras instead of horses, doing that extra check-up to resolve the issue.

#### Four clusters of presence

By adding the voices of the system and the profession, maxims of presence can be dichotomised as either *being an asset* (loyal) or *seeing the essential* (unfettered), and as either *passionate* (idealistic) or *humble* (pragmatic).

##### Institutionalise (methodical presence: be an asset passionately)

Undeterred by any extra work that this might require, the GP seeks to harmonise methodical, institutionally sanctioned practice with attentiveness to the quirks of the situation,

- taking the initiative to develop structures that facilitate cooperation;
- “reeling in” patients who are at risk of being let down by the system;
- persuading patients to accept medically rational explanations for their symptoms or side effects of treatments; and
- going to great lengths to get everything done, speeding up or working late as necessary.

##### Understand (autonomous presence: see the essential passionately)

The GP tries to see clearly the other’s concerns, regarding it unimportant to follow standard approaches or force the other to accept what is medically true. For instance, the GP

- encourages honesty and openness by taking on an unthreatening, unimposing, and uninquisitive demeanour;
- invests the extra time needed to reach a well-grounded decision although time constraints might be thereby violated;
- tries to remain open intellectually to impressions that would require reinterpreting the problem; and
- stays connected to the patient also when powerless to relieve their suffering.

##### Embrace (contributing presence: be an asset humbly)

The GP strives to meet the demands of the other without transgressing the bounds of the institution. Adapting to the socially expected, the GP

- carries out menial tasks or manages complicated situations without assistance instead of bothering co-workers;
- interrupts what they are currently doing in order to assist someone else;
- dabbles in matters that they do not fully master in order to compensate for the failings of other healthcare professionals;
- does as best they can with tools that barely work, instead of demanding replacements or upgrades; and
- accepts, out of loyalty, new routines although they would seem to bring no clear benefits.

##### Accompany (creative presence: see the essential humbly)

The GP plays the part of fellow human being rather than authority, allowing idiosyncratic demands rather than bureaucratic or idealistic agendas to direct their attention. Possibly at some personal cost, the GP

- prescribes medications or commences investigations that cannot be generally recommended;
- writes a sick note out of concern for the other’s well-being rather than focusing on legal aspects;
- allows the patient to voice yet another concern even though time is running short; and
- avoids causing unnecessary anxiety by playing down or keeping silent about aberrant but presumably benign findings.

#### Four clusters of control

Maxims of control can be dichotomised as either *exerting authority* (loyal) or *calling the shots* (unfettered), and as either *conservative* (idealistic) or *secure* (pragmatic).

##### Command (methodical control: exert authority conservatively)

The GP decides what is or is not worthy of their time or attention. Upholding professional virtues while protecting their working environment, the GP

- conceives of interprofessional teams as win–win arrangements because of reduced responsibility and saved time slots;
- diverts responsibility for “fixing” lifestyle-related health issues by turning them around to the patient as advice or “home assignments”;
- rejects excessive demands for investigations or prescriptions by appeal to rules and routines; and
- reduces today’s to-do list by postponing medically less important issues.

##### Shield (autonomous control: call the shots conservatively)

In order to protect the time and space that they need to work conscientiously, the GP rejects demands that have a consumerist tinge or cast them in the role of servant. Some friction is produced when the GP refuses to

- interrupt consultations to respond to requests for immediate assistance;
- act as go-between by writing referrals to one specialist on the behalf of another;
- prescribe drugs in bad faith or order futile investigations; or
- trudge on without enough time to breathe and reflect.

##### Playact (contributing control: exert authority securely)

The GP grabs low-hanging fruit, gaining goodwill while avoiding personal responsibility for what is sacrificed. For instance, the GP will

- bring straightforward matters to a close as quickly as possible, thus saving time and resources;
- let the patient decide what matters to discuss and what procedures to undertake whenever the system promotes such an approach; and
- defend the system when it fails to prioritise patients with greater medical need.

##### Survive (creative control: call the shots securely)

Caught between the imperatives of doing what is right, expected, and advantageous, the GP aims to survive the onslaught. Turning their back to demands from all three voices external to the self, the GP will

- play for time by suggesting pseudo-solutions or procrastinating when a patient’s requests are becoming too taxing; and
- renegotiate duties or refuse outright when demands become too onerous.

#### Four clusters of disinterest

Finally, maxims of disinterest can be dichotomised as either *working up the case* (loyal) or *straightening it out* (unfettered), and as either *austere* (idealistic) or *prosaic* (pragmatic).

##### Master (methodical disinterest: work up the case austerely)

By stepping back from the encounter, the GP seeks mastery over its generalisable features. With cool scientific curiosity, the GP

- uncovers the truth behind the matter, preferably with a diagnosis as proof of success;
- disabuses the other of whatever misconceptions or irrational beliefs they may hold;
- labours conscientiously to reduce medical risk, at the cost of personal discomfort if necessary; and
- deflects medically unreasonable demands by reference to guidelines or tradition.

##### Focus (autonomous disinterest: straighten it out austerely)

The GP speaks confidently with the voice of the profession. Unbound by the imperative to follow particular procedures, they hope to gain an unobstructed view of the object by

- relying on their clinical judgment in deciding what investigations can be put on hold or forgone entirely, thus avoiding being sidetracked by endless lists of possibilities;
- giving little thought to directives that impede conscientious practice;
- emphasising the need to develop certain core competencies that are unique to GPs; and
- being happy to leave – contrary to the system’s ideal of comprehensiveness – cases that require narrow or specific skill sets in the care of specialists.

##### Finesse (contributing disinterest: work up the case prosaically)

The GP values consensus among peers, preferring to do workups as prescribed by guidelines and policy decisions, but is also sensitive to unwritten rules and to the system’s overarching pursuit of efficiency. The GP therefore

- propagates their own mindlines by handing out titbits of wisdom or “shooting from the hip”; and
- screens for disease or relieves the other’s suffering through routine interventions.

##### Withdraw (creative disinterest: straighten it out prosaically)

Rather than listening to any of the four voices, the GP recognises as futile many lines of action that others find worthy of pursuit. For instance, the GP

- accepts matter-of-factly the risk of making medical mistakes;
- shrugs at improvement projects that may look good on the paper but hold no real promise; and
- lets patients hold their irrational beliefs rather than trying to convince those that do not want to be convinced.

## Discussion

In this paper, we have presented a conceptual framework and a sub-process that explain the often-conflicting ethical demands that GPs experience in their daily work. We have deliberately turned away from technical and topic-specific aspects, looking instead for abstract ethical principles, values and virtues. After considering the strengths and weaknesses of our study, we will conclude this paper by reading some substantive theories on GPs’ ethics in the light of our findings.

### Strengths and weaknesses

Embracing the pragmatist roots of Grounded Theory, we believe that the value of this study is ultimately decided by whether it helps the reader to see more clearly what GPs do and why. That said, we will presently consider its methodological strengths and weaknesses, and thereafter turn to those that ensue from its scope, content, and level of abstraction.

### Methodological considerations

#### Recruitment and sampling

While we do not claim “representativeness” of the Swedish primary care context by any quantitative standard, we believe that our material covers most contextual extremes that affect the main concerns of GPs. While snowballing carries some risk of introducing homogeneity, in this case it allowed us to investigate and understand better the importance of individual factors versus context.

#### Technical literature

To give the data an opportunity to “speak for itself” without immediately polluting it with existing theories, we postponed most review of technical literature. Because the theories with which we were already familiar have a considerably higher level of abstraction, we do not believe that they have overly distorted our analysis.

### Scope and content

A considerable strength of our study is its multi-dimensional model of ethical decision making which, together with its high level of abstraction, brings out the complexity of GPs’ practice. Speaking in terms of maxims of action reveals tensions that would easily remain invisible in a more concrete account of GPs’ “behaviour.” For instance, refusing to prescribe certain inappropriate drugs might be described as *methodical* (loyal idealistic) in one context but *autonomous* (unfettered idealistic) in another depending on the expectations of peers and other staff. Although at first glance the action appears to be the same, its symbolical connotations (and perhaps, consequences) are vastly different.

Compared to similar studies, this study is thematically less focused as it considers the everyday practice of GPs as a whole rather than a specific set of encountered issues. This can be conceived of as either a strength or a weakness. On a positive note, we have been able to discern patterns that run across activities and themes. The framework stays true to the data by emphasizing factors that are operative in ethical decision making in general, yet are easily recognisable in particular cases: idealism, social pressure to conform, need for self-protection, unequal distribution of power and suffering, and so on. The trade-off has been, as for any middle-range theory, a reduced capability for discovering responses to specific problems and conditions that modify such responses. Our conceptual framework might also appear too abstract to readers who are not already familiar with concrete problems in general practice. We believe, however, that using it as a framework through which findings in substantive theories can be interpreted will yield deeper insights into the ethics of GPs.

Although our study is limited to GPs, we cannot exclude the possibility that its results are transferable to other physicians or even other healthcare professionals. However, because hospital-based physicians may not be committed to a holistic approach, the imperatives voiced by the situation and the system are bound to differ from what we see here. Much the same can be said about other healthcare professionals because of the different roles that they inhabit within the system.

### Relation to other works

Terming the sources of different norms “voices” is an idea that we owe to Hansen [18]. His model is a two-dimensional one, with the voices of the system and the matter (in Swedish, “saken”) on opposite ends of one axis, and those of the profession and the person on the other. Despite the similarities between Hansen’s framework and ours, their differences run deep. Most crucially, Hansen’s model excludes a priori the possibility of most of the interactions between voices that we present in this paper.

Our finding that virtue plays a role in the practice of GPs resonates with those in other works, such as in the study by Braunack-Mayer [13]. In our framework however, the concept of virtue is more abstract and its influence less specific. This is because the voice of the profession, as we understand it, is essentially void of context; the contextual counterpoint is instead picked up by the voice of the system. As we have seen, the latter asserts many values cherished within the profession, but also some that concern its own continued existence. The case of “continuity” is a case in point. From the perspective of the voice of the profession, commitment to a patient over time is unambiguously good because it increases the GP’s idiographic knowledge, allowing them to make better decisions over time. The system might also come to value continuity, but given its plurality of values, it will do so only to the extent that continuity promises to be an efficient means to its ends, all things considered. Implementation of new evidence is another cause of contention. It is clearly a main interest of the system, the perpetuity of which depends not only on delivering goods but also on conveying an image of progress. In contrast, the voice of the profession is more conservative, impelling the GP to disavow interventions that lack evidence, but rarely to impose upon the patient even evidence-based ones.

GT research on GPs’ decision making has been mainly limited to substantive theories, albeit ones that yield important insights. In this last section, we will comment on some of them, providing examples of how they can be read through our framework. We hope to show how our multi-dimensional view of GPs’ ethics can be used to compare events across topics and activities, aiding transferability of results from substantive theories. We have selected from them four emergent themes, each of which corresponds to a conflict between two or more voices. First, there is the *disappointment in oneself* that GPs experience when acting in bad faith to meet the demands of the situation and the self. Second, demands voiced by the system are sometimes resisted in order to *protect the self*. Third, and perhaps most controversially, *standing up to the system* can sometimes be motivated by appeal to professional ideals. Last, we shall see the perils of detachment when used to *circumvent disruptions* to rational decision-making.

### Professional virtue and disappointment in oneself

Several substantive theories highlight the pressures that divert GPs’ actions from their perceived ideal course. For instance, Henriksen and Hansen vividly describe how GPs found themselves in “a constant state of readiness” because of pressures to “prescribe in a way that threatened their self-image,” and were frequently disappointed in themselves for failing to live up to their ideals in that respect [19]. Through these encounters runs a note of self-consciousness – triggered by workload, time constraints, and uncertainty caused by diverging ideals, “bombardment” from pharmaceutical companies, and patients that “crawl under your skin” – that restricts possible responses to the clusters of *vigilance* or *control.* Furthermore, the demands of the profession and the demands of the system are all but monolithically aligned and stand in direct opposition to responsiveness to the patient’s plight. Ultimately, the GP faces the binary choice between “digging in their heels,” which we interpret as aiming to *command* (methodical control), or “giving in,” which would be to *suffice* (creative vigilance). What becomes particularly salient through the lens of our framework is how their options were severely restricted beforehand, first by forced compliance with one voice, and then through a tripartite deadlock.

### Protecting the self

In the study by Bower et al., many GPs assigned higher priority to the patient’s agenda than to meeting specific performance targets [7]. Clearly, doing so did not quite relieve the strain; there were signs of *control* in how the GPs relied on an “additive-sequential” consultation model that allowed them to defer some decisions to later appointments. As a trade-off, more complex issues were less effectively handled. All in all, the maxim behind the preferred approach gravitated towards *surviving* (creative control).

In the study by Baik et al., GPs who recognised that a patient was depressed spent considerable time “selling” this diagnosis in a way that the patient might agree with [20]. Their need for self-protection was evident in their hesitation to “open the door” because of the lengthy negotiations that would surely ensue. This choice resulted in detachment and lack of candour. All in all, the maxim can be identified as a *playact* (contributing control).

### Standing up to the system

Professionally motivated resistance to the system is seen in the findings of Müller-Riemenschneider et al. [8]. Here, a standardised procedure was rejected not primarily because of time constraints or other self-related concerns, but because of its limited utility and expected adverse effects on the doctor–patient relationship. A similar stance was found by Agarwal et al. among GPs who, when deciding whether or not to prescribe insulin to older people with type 2 diabetes, would take into consideration not only medical facts but also the patient’s situation, background, personality, and fears [21]. Interestingly, the authors seem to consider this a deficiency that could be remedied through education and specialist support. As we see it, a case could easily be built in defence of the maxim that prevailed in both cases – *understanding* (autonomous presence) – on the grounds that attentiveness to contingencies and healthy dose of scepticism is reasonable in situations of uncertainty.

### Circumventing disruptions

Finally, we must consider the potentially pernicious union of loyalty and detachment. In their study on shared decision making in diabetes care, Shortus et al. found that the views of informants ranged from “treating to target” (exhorting the patient when necessary) to “personalized care” (prioritising the patient’s right to decide) [22]. A similar polarisation is heard in the accounts retold by Freeman and Sweeney [1]. Concerned with the effects on the patient’s life as a whole, these GPs emphatically alienated themselves from the “evidence based mafia” that would treat “diseases rather than patients.” Rather than dismissing such concerns as irrational “resistance to evidence,” we believe that one should seek a deeper understanding of the underlying conflict of values. A key is provided by Halpern and Little, who describe how clinicians, intent on saving lives, tend to try hard to “circumvent the disruptions that interfere with rationality.” [23] Such efforts to, in our vocabulary, *master* (through methodical disinterest) the other may potentially “shade into a tendency to regard others as objects to be manipulated rather than agents to be respected,” which we would describe as *finesse* (contributing disinterest). Given their continual relationships with many patients, it is no surprise that GPs are found among the ones who raise their voices in protest against such maxims.

## Conclusions

GPs’ ethical decision-making can be conceived of as choosing a maxim of action while under pressure from competing sets of norms, or “voices.” As a step towards a middle-range theory on quality from the perspective of GPs, we have presented in this paper a four-dimensional conceptual framework that explicates the possible interactions between those voices. Our framework addresses performative questions such as “Why do GPs fail to implement guidelines?” by explaining the nature of the ethical conflicts that they experience and showing how not mere causes, but actual *reasons* for such choices arise from the ethical complexity of their work. We thus provide some clues as to why efforts to improve quality by imposing additional norms may meet with varying degrees of success.

## Abbreviations

EBM: Evidence Based Medicine
GP: General Practitioner
GT: Grounded Theory
